# Effectiveness of community-based football compared to usual care in men with prostate cancer: Protocol for a randomised, controlled, parallel group, multicenter superiority trial (The FC Prostate Community Trial)

**DOI:** 10.1186/s12885-016-2805-0

**Published:** 2016-10-03

**Authors:** Eik Bjerre, Ditte Marie Bruun, Anders Tolver, Klaus Brasso, Peter Krustrup, Christoffer Johansen, Robin Christensen, Mikael Rørth, Julie Midtgaard

**Affiliations:** 1 University Hospitals Centre for Health Research (UCSF), Rigshospitalet, University of Copenhagen, Copenhagen, Denmark; 2 Department of Mathematical Sciences, Faculty of Science, University of Copenhagen, Copenhagen, Denmark; 3 Copenhagen Prostate Cancer Center, Department of Urology, Rigshospitalet, University of Copenhagen, Copenhagen, Denmark; 4 Department of Sports Science and Clinical Biomechanics, SDU Sport and Health Sciences Cluster (SHSC), University of Southern Denmark, Odense M, Denmark; 5 Sport and Health Sciences, College of Life and Environmental Sciences, University of Exeter, Exeter, UK; 6 Department of Oncology, Rigshospitalet, University of Copenhagen, Copenhagen, Denmark; 7 Unit of Survivorship, Danish Cancer Society Research Center, Copenhagen, Denmark; 8 Musculoskeletal Statistics Unit, The Parker Institute, Bispebjerg and Frederiksberg Hospital, Copenhagen, Denmark; 9 Department of Public Health, University of Copenhagen, Copenhagen, Denmark

**Keywords:** Prostate cancer, Soccer, Exercise, Physical activity, Quality of life

## Abstract

**Background:**

Prostate cancer is the most common non-cutaneous malignancy in men. Today most patients may expect to live years following the diagnosis and may thus experience significant morbidity due to disease progression and treatment toxicity. In order to address some of these problems exercise has been suggested and previously studies have shown improvements of disease specific quality of life and a reduction in treatment-related toxicity. Cohort studies with long term follow up have suggested that physical activity is associated with improved survival in prostate cancer patients. Previously one randomised controlled trial has examined the efficacy of football in prostate cancer patients undergoing androgen deprivation therapy to usual care and reported positive effects on lean body mass and bone markers. Against this background, we wish to examine the effectiveness of community-based football for men diagnosed with prostate cancer.

**Methods:**

Using a randomised controlled parallel group, multicenter, superiority trial design, two hundred prostate cancer patients will be recruited and randomised (1:1) to either community-based football one hour twice weekly or to a control group. The intervention period will be six months. The primary outcome is quality of life assessed after 12 weeks based on the change from baseline in the Functional Assessment of Cancer Therapy–Prostate questionnaire. Secondary outcomes are change from baseline to six months in quality of life, lean body mass, fat mass, whole body and regional bone markers, as well as physical activity and functional capacity at 12 weeks and six months. Safety outcome variables will be falls resulting in seeking medical assessment and fractures during the six-month period.

**Discussion:**

Football is viewed as a case for non-professional, supervised community-based team sport for promoting long-term physical activity in men diagnosed with prostate cancer. This randomised trial will provide data on effectiveness and safety for men with prostate cancer when football training is delivered in local football clubs.

**Trial registration:**

Clinicaltrials.gov identifier: NCT02430792

## Background

Prostate cancer (PCa) is the most frequently diagnosed non-cutaneous cancer in men worldwide [1]. In comparison to most other malignant diseases, PCa progresses slowly. Presently more than 90 % of all cases of PCa are detected early, with the 10 and 15-year relative survival rates at 98.8 and 94.3 %, respectively [1]. However, treatment of PCa is often associated with side effects and as the patients are likely to survive many years following diagnosis and primary treatment, quality of life (QoL) is of growing interest. PCa patients managed with hormonal therapy face an elevated risk of becoming overweight and for developing metabolic syndrome, type 2 diabetes and cardiovascular disease [2], which regular physical activity may counteract. Despite this fewer than half of men living with PCa follow the official physical activity guidelines [3]. Moreover, unlike many other cancer patient groups, PCa patients do not change their health behaviour spontaneously after being diagnosed [4]. Cohort studies examining the relationship between physical activity and disease progression and survival in men with PCa have shown that vigorous activity is associated with better outcomes [5–7].

Recent systematic reviews of exercise intervention trials in men with PCa indicate that exercise has a beneficial effect on muscular fitness, cardio-respiratory fitness, functional task performance, lean body mass (LBM), fatigue and QoL [8–10]. Furthermore exercise has been shown to meet the requests male cancer patients have for active, rational activities [11, 12]. The vast majority of existing trials, however, have examined the effects of hospital-based, short-term and cost-intensive exercise interventions with a short follow-up period and low degree of adherence [13]. Therefore the long-term effects and applicability of interventions promoting physical activity remain undocumented [14].

Participation in sport is acknowledged as important for public health [15]. The most common sport for men in Denmark and many other countries is football [16]. Growing evidence suggests that recreational football, i.e. non-tournament-based small-sided games, can have significant health promoting effects in various populations [17–19]. It has also been shown that football may provide peer-to-peer psychosocial support and improve social capital [20], potentially increasing long-term adherence. Therefore, we recently examined the efficacy of recreational football in men with PCa undergoing androgen deprivation therapy (ADT). The results show that the football training improved LBM, muscle strength and markers of bone strength [21, 22] and that men experience football as an opportunity to regain control and responsibility for their own health without being in the role of patient [23].

Usual care for men with PCa in Denmark is rehabilitation (often aerobic and/or strength training) offered by local authorities. However, substantially fewer men than women (30 vs. 70 %) participate in rehabilitation [24], indicating that rehabilitation efforts might should be gender-oriented [25]. In this study, the participants in the control group are informed of the official physical activity guidelines and advised to continue their daily living, as they normally would do, not guiding them to other interventions neither preventing them to do so.

Thus, we will examine if recreational football, delivered in local football clubs (i.e. community-based), may complement existing rehabilitation approaches by promoting adherence to exercise and improving QoL and physiological health measures.

## Objective

The primary objective of the FC Prostate Community Trial (FCPC) trial is to determine whether community-based football is superior to usual care for improving cancer-specific QoL after 12 weeks of participation.

The secondary objectives are to determine whether community-based football is superior to usual care for improving PCa-specific QoL, lean body mass, fat mass, bone mineral density and bone mineral content after six months and functional well-being and physical activity after 12 weeks and after six months. The safety of the intervention will also be evaluated based on falls resulting in seeking medical assessment and fractures after six months.

## Methods

### Trial design

The FCPC Trial is a randomised controlled parallel-group superiority trial with two parallel groups (community-based recreational football and usual care) that examines QoL after 12 weeks as the primary endpoint. Participants will be randomised (1:1) and the trial design is pragmatic [26].

### Participants

Patients age 18 years or older diagnosed with PCa, able to read and write Danish and willing to sign an informed consent are eligible for participation in the study. Patients cannot be included if they have undergone prostatectomy within six weeks prior to participation, are prohibited from participating in football training by their primary physician or have a hip or spine BMD T-score lower than −2.5 (i.e. the criterion for osteoporosis). Table 1 lists the eligibility criteria.

**Table 1:** Eligibility criteria

Inclusion	Exclusion
• Patients diagnosed with prostate cancer • Age: ≥18 years • Able to read and complete questionnaires in Danish • Signed informed consent	• < 6 weeks prostatectomy • Football training not allowed by primary physician • Hip or spine bone mineral density < −2.5 T-score

**Fig. 1:**
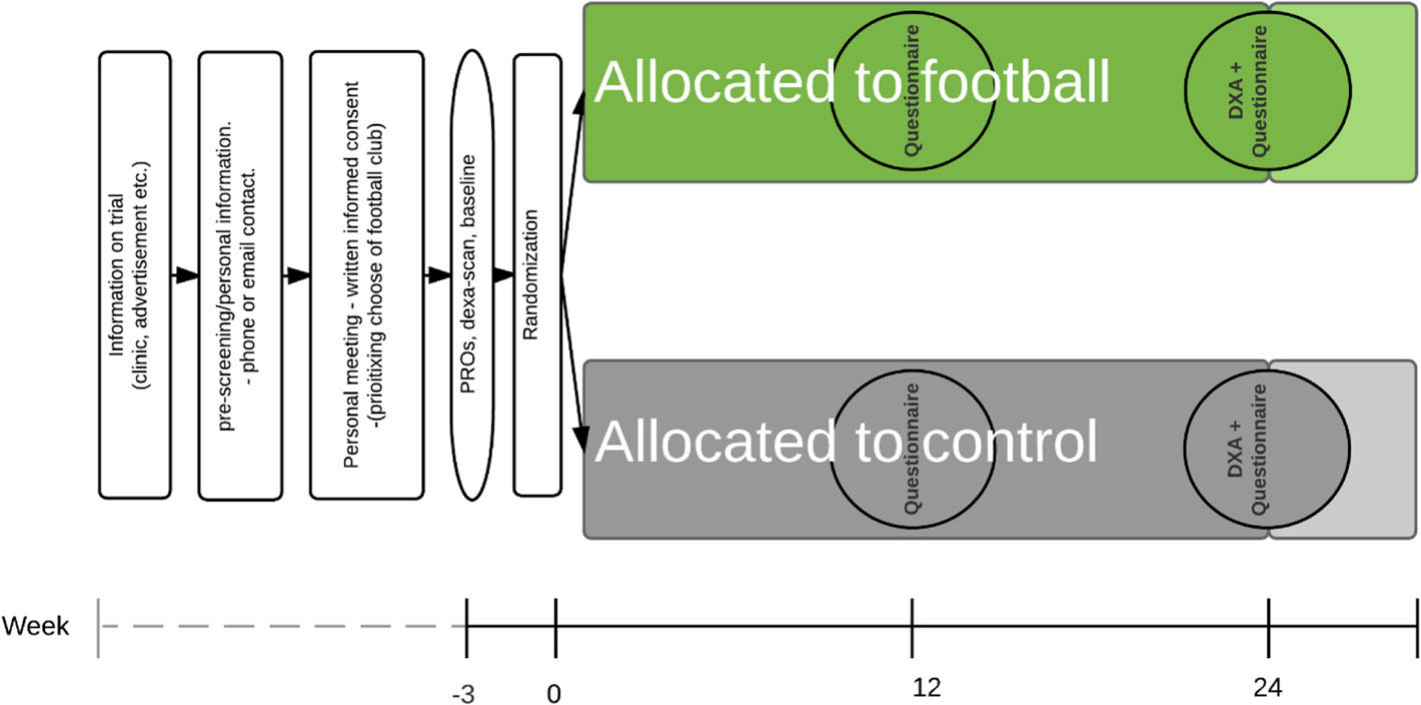
Trial design and timeline

Patients will be recruited from urological clinics, which will provide information on the study and promote patients to participate in the study. Figure 1 presents a timeline for participation and the trial design.

Study sites and recruitment status are available at clinicaltrials.gov under trial registration number NCT02430792.

### The football intervention

**Fig. 2:**
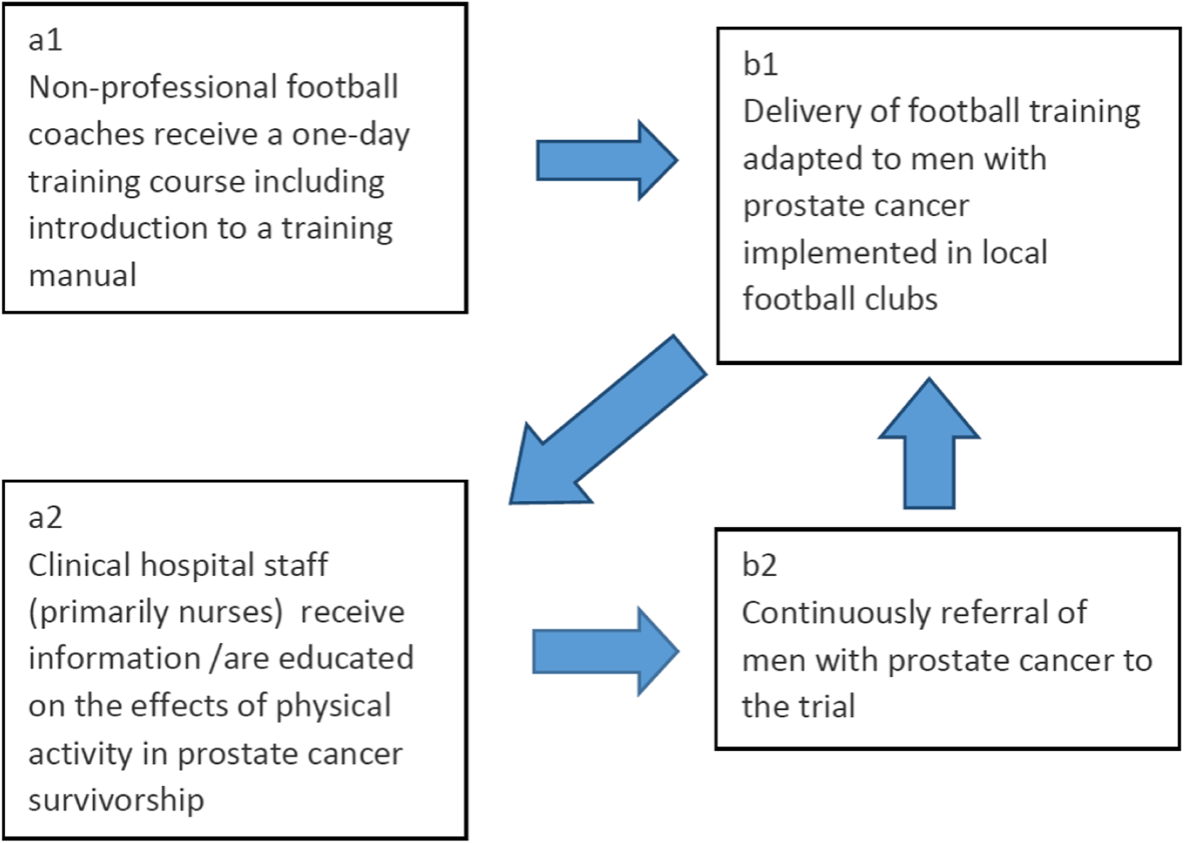
Action theory. The trial involves two major activities: (a1) education/training of non-professional football coaches recruited from local sports clubs delivering the intervention and (a2) education of clinical hospital staff (primarily nurses) with the authority to refer prostate cancer patients to supportive care interventions. These two major activities are expected to produce: (b1) delivery of community-based football training adapted to men with prostate cancer and (b2) continuous referral of men with prostate cancer to the trial. As still more men with prostate cancer are expected to be referred from the clinic, the assumption is that the number of men with prostate cancer participating in community-based football will increase (b2 → b1). The expectation is that these men will share what they think of the intervention with the medical team/staff and as such contribute to the further education of the clinical staff (b1 → a1)

**Fig. 3:**
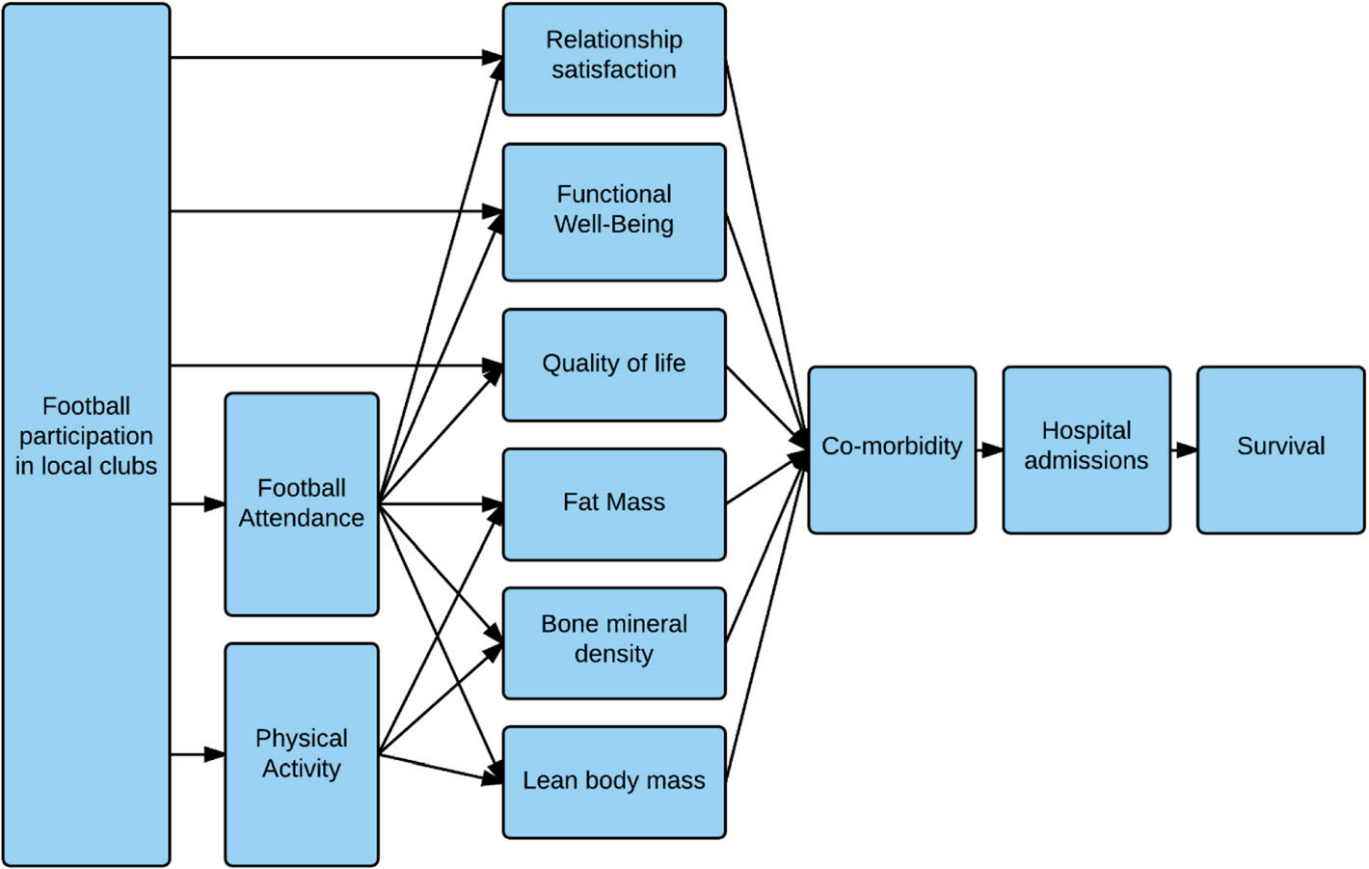
Assumed causal pathways

Participants allocated to the intervention group will be offered one hour of recreational football twice weekly. The football training sessions consist of a 20-min warm-up based on the FIFA11+ concept, though modified to suit older players [27]. Next, participants will spend 20 min practising dribbling, passing and shooting. The training sessions will then end with 20 min of 5–7-a-side football. Two coaches recruited from the local football club will be in charge of all training sessions. The coaches are expected to have experience as either a player or coach but no other formal qualifications are required. The coaches will be required to have passed a first-aid course and to complete a 10-h course involving lectures on PCa treatment, patient experiences of PCa and a manual describing the content of the training, including the FIFA 11+ concept intended to prevent injuries. Participants will be told to avoid hard tackles and other actions that carry a risk of injury. In the event of injuries participants will remain in their allocated group and will be encouraged to participate in football practice again after recovery. Adherence to the intervention will be recorded by the coaches. Participants allocated to football training will be able to track their individual adherence and compare it to an average adherence rate of participants in the football group. A logical model, presented in Table 2, summarises the key inputs, activities and intended outputs of the intervention, while Fig. 2 presents the activities and Fig. 3 shows the assumed causal pathways for the effect of the intervention on the individual.

**Table 2:** Logic model: The FCPC Trial

Inputs (resources)	Activities	Outputs	Outcomes (short-term)	Impacts (long term)
Financial resources • Funding Human resources • Urologist and urological nurses • Local experienced football coaches Products • Football-training manual • Disease specific football-coach education • Tablet based app to register attendance and field-test performance	• Collaboration with local football clubs • Education of local coaches • Collaboration with urological clinics • Information to clinical staff and patients' on the possibilities of referral for the study • Provision of feedback on attendance and progress (field tests)	• Prostate cancer patients referred to and participating regularly in football training	Improved: • Quality of life • Physical activity level • Fat mass • Muscle mass • Bone mineral density and content • Functional well-being • Dyadic adjustment	Improved: • Survival Reduced: • Co-morbidities • Hospital admissions • Medication usage
Planned work		Intended Results

### Control group

Participants allocated to the control group will receive a phone-based counselling session (5–15 min) as part of the information on group allocation, as well as information via email on the current physical activity guidelines.

### Outcomes

The *primary outcome* is change in PCa-specific QoL from baseline to 12 weeks measured with the fourth version of the Functional Assessment of Cancer Therapy–Prostate (FACT-P) questionnaire [28], a self-reported, multidimensional QoL instrument specific for PCa patients [29]. The questionnaire consists of 27 general cancer items covering four domains - physical, social/family, emotional and functional well-being - supplemented with 12 specific PCa-related items. All items are scored on a 0–4 Likert scale. The total FACT-P score ranges from 0 to 156, higher scores indicating higher QoL.

Table 3 presents all outcomes with specified measurement variable, analysis metric, method of aggregation and time point.

**Table 3:** Outcomes with specified measurement variable, analysis metric, method of aggregation and time point

Concept	Specific measurement variable	Patient- level analysis metric	Method of aggregation	Time point(s)
Primary outcome				
Quality of life	Functional Assessment of Cancer Therapy - Prostate Questionnaire	Change from baseline	Mean	12 week
Secondary outcomes				
Quality of life	Functional Assessment of Cancer Therapy - Prostate Questionnaire	Change from baseline	Mean	6 month
Muscle mass	Whole-body lean body mass	Change from baseline	Mean	6 month
Fat mass	Whole-body fat mass	Change from baseline	Mean	6 month
Whole body bone strength	Whole-body bone mineral content	Change from baseline	Mean percent	6 month
	Whole-body bone mineral density	Change from baseline	Mean percent	6 month
Self-reported physical activity	International Physical Activity Questionnaire	Change from baseline	Mean	12 week and 6 month
Functional well-being	Subscale from Functional Assessment of Cancer Therapy-Questionnaire	Change from baseline	Mean	12 week and 6 month
Regional bone strength	Lumbar spine bone mineral density	Change from baseline	Mean percent	6 month
	Femoral neck bone mineral density	Change from baseline	Mean percent	6 month
	Total hip bone mineral density	Change from baseline	Mean percent	6 month
Safety	Participants with any fracture	Number in each group	Proportion	6 month
	Participants with falls that resulted in seeking medical assessment	Number in each group	Proportion	6 month
Exploratory outcomes				
Dyadic adjustment	DAS-7	Change from baseline	Median	12 week and 6 month

The 12-Item Short Form Health Survey (SF-12) and EQ-5D-5 L, a standardised instrument by EuroQol for use as a measure of health outcome, will be collected for use in later economic evaluations of the intervention.

### Sample size and power considerations

The sample size calculation is based on the detection of a minimal clinical important difference between groups on six points on the FACT-P questionnaire [28] at 12 weeks with a standard deviation of 15 FACT_P points. A two-sided significance level of 5 % and power of 80 % were chosen; The standard deviation is based on a previous exercise trial [30] but increased slightly as FCPC participants will be more heterogeneous than the participants in that trial. Consequently, 100 participants will be needed in each group, i.e. 200 participants in total, in order to detect a statistically significant difference between groups. No interim analysis will be performed. However, if the anticipated sample size has not been enrolled by 1 May 2017, recruitment will end and final analyses will be performed with the number of patients recruited at that point.

### Randomisation

*Sequence generation*, the allocation of participants, will be done using a computer-generated list of random numbers. The allocation will be stratified by center and by androgen deprivation status (yes/no) with a 1:1 allocation using random block sizes. A statistician not involved in the trial uploaded all the allocation sequences in the trial management system. The eligibility of participants will be assessed by either investigators or project nurses at each center. The randomisation will be performed centrally by researchers at the University Hospitals Centre for Health Research, Copenhagen University Hospital, Rigshospitalet.

*Allocation concealment* will be implemented, as the allocation sequence will be hidden from the researchers and, using web-based trial management, no one other than the statistician who uploads the random number lists have access to the lists.

### Blinding

Lean body mass, fat mass, bone mineral density and bone mineral content outcomes measured with dual-energy X-ray absorptiometry (DXA) at six months will be performed by an external assessor blinded to group allocation. It is not possible to blind participants or staff due to the nature of the intervention.

The analyses will be performed with the group identity blinded and the groups denoted as A and B. Blinded analyses of both the primary and secondary outcomes will be presented to the research group and decisions on the abstract and the conclusion for the main publication will be made before revealing group identity.

### Data collection

A web-based trial data management system (easytrial.net) will be used to collect data on the primary outcome, FACT-P and the other self-reported outcomes (International Physical Activity Questionnaire (IPAQ), seven-item Dyadic Adjustment Scale, SF-12 and EQ-5D-5 L), in addition to demographic characteristics at baseline. The web-based trial data system will distribute questionnaires and invitations to DXA scans according to the date for randomisation. The trial data system will send a unique e-mail to each trial participant’s personal e-mail address. Applied to both groups, this procedure will ensure that responses are unaffected by an interviewer or changing conditions. Participants will be asked to complete questionnaires 12 weeks and six months after randomisation, and come to a six-month DXA assessment, even if they discontinue the allocated treatment. Non-adherence to the allocated intervention is thus not necessary off study, and participants will still be encouraged to do follow-up assessments.

DXA scans will be used to collect data on body composition: LBM and fat mass, and bone markers: bone mineral content (BMC) and bone mineral density (BMD). Data accuracy will be ensured, as participants will add questionnaire responses directly into the trial data system, while DXA outcomes extracted from DXA scanners will also be directly added into the trial data system. The trial data system adheres to the International Council for Harmonisation Guideline on Good Clinical Practice (ICH-GCP) and the Danish data protection legislation. Figure 1 provides time points for data collection, study visits, enrolment, intervention and assessments.

### Statistical methods

#### Plan for statistical analyses

The primary statistical analysis targets the effect on PCa specific QoL of a treatment policy offering community-based football to men with PCa. In particular, patients will be analysed in the treatment group to which they were randomly allocated according to the intention-to-treat principle. The appropriate method for addressing this effect will depend on the assumptions made about missing data (dropouts). Our main analysis is valid under the missing at random assumption but we will also present sensitivity analyses robust to non-ignorable patterns among patients with incomplete data.

Per protocol analyses will be performed to estimate the de jure effect of the treatment for compliant patients. The per protocol population will be defined as intervention group participants who attended the football intervention at least 12 times in the first 12 weeks and 24 times in the six-month intervention period.

Significance tests will be two-sided with a maximal type I error risk of 5 %. To address the problem of multiple comparisons for secondary analyses when several outcomes are tested or multiple constracts are extracted from the same statistical model, p-values will be adjusted using the step-down Bonferroni method of Holm [31] or appropriate modern alternatives.

#### Trial profile

A CONSORT diagram will show trial participant flow. The number of screened patients, the patients who meet the inclusion criteria and trial subjects included in analyses will be reported together with reasons for exclusion of trial subjects.

#### Primary outcome

The continuous FACT-P outcome score will be calculated using the official scoring guideline. As described in the scoring guideline missing items will be prorated by multiplying the sum of the subscale with the number of the items in the subscale, then divided by the number of items answered. This will be done if more than 50 % of the items are answered in the subscales and 80 % are answered in the total questionnaire. The change score of the total FACT-P at 12 weeks will be calculated by subtracting the total 12-week score from the respective trial participant’s baseline score. Analysis of covariance will be used [32], group and ADT status will be set as factors, the response will be change in FACT-P and covariates will be age and baseline score. The results will be presented as least squares means (LSMEANS) differences between the two groups with 95 % confidence intervals and *p*-values.

#### Secondary outcomes including safety outcomes

Changes from baseline to six months for LBM, BMD, BMC and total body fat mass will be analysed in the same manner as the primary outcome.

QoL, functional well-being and physical activity (based on metabolic equivalent of task values derived from IPAQ) measured at baseline, 12 weeks and six months will be analysed using a mixed model for repeated measurements. Changes from baseline to 12 weeks or six months will be treated as the response. The model will include fixed effects of factors: group, ADT, sampling time and their interactions, and the analysis will be adjusted for age and baseline value. The correlation between measurements on the same participant will be modelled using a random effect of participant.

Safety outcomes will be listed for each group and the number of falls that resulted in seeking medical assessment and fractures will be compared across groups using Fisher's exact test.

Subgroup analyses will be reported for the patients treated with ADT, which means that results will be given for both the overall treatment effect and for the subgroup obtained by stratifying according to ADT. To verify the credibility of our subgroup analyses, we will apply the criteria proposed by Sun and colleagues [33], i.e. the subgroup variable is a baseline characteristic, a stratification factor, specified a priory and includes only a small number of analyses.

#### Outline of figures and tables

The first figure in the main publication will be a CONSORT diagram and the second figure will illustrate changes in the primary and secondary outcomes, with the exception of safety outcomes, at 12 weeks and six months, according to treatment group.

A third figure will display mean curves for the primary outcome for participants in different groups according to the pattern of missing data. In particular, mean curves will be shown separately for completers and participants with missing data at one or more assessment times. The figure will be used to guide the type of sensitivity analyses performed to adjust results for a potentially deviating pattern for patients with incomplete observations.

The main publication will also include three tables, one delineating the characteristics of trial subjects, one showing changes in primary and secondary outcomes at 12 weeks and at six months, and one presenting safety outcomes according to group and type.

#### Ethics

The Ethics Committee for the Capital Region of Denmark (file number H-2-2014-099) and the Danish Data Protection Agency has approved the trial and the trial is registered at clinicaltrials.gov: NCT02430792.

Any changes to the protocol that influence the conduct of the study, affect the safety or benefits for participants, i.e. study objectives, study design, eligibility criteria and study procedures, will be documented in protocol amendments, which must be approved by the Ethics Committee for the Capital Region of Denmark and which will be reported when the study is disseminated.

Patients will receive written and oral information about the study. Project nurses or researchers with suitable training will obtain informed consent based on the standard operating procedure. Consent will be obtained prior to any study activities and study participants can withdraw from the trial at any time without any explanation or consequences. Although aware that they are under no obligation to provide an explanation, individuals who withdraw will be asked why they chose to discontinue the trial.

## Discussion

The FCPC Trial is a randomised, controlled, parallel-group trial examining the effectiveness of community-based football on QoL in men with PCa.

Whether exercise should be an integral part of treatment for PCa is under debate [34, 35]. The effects of exercise on side effects of treatment has been shown [36, 37]. However, previous exercise trials for men with PCa reporting positive effects had short follow ups and took place in clinical settings, thus not utilising community-based structures for sport and exercise.

The FCPC Trial will provide empirical data on whether local sport clubs are possible venues for clinical health promotion through a team-based exercise intervention. With experience from this study, we hope to gain further insight into the effects in a real-world setting and, through subgroup analyses, confirm findings from a previous study on PCa patients undergoing ADT [21].

Since numerous men currently live many years with PCa, it has been recommended that the disease be considered similar to other chronic diseases [38]. With this in mind, issues affecting QoL are of primary concern, not just for the patient but also for the treating urologist, who is often the primary treating physician [39]. We argue that football training is a supportive intervention where short-term QoL is of primary concern both for the patient and in the context where long-term adherence is a prerequisite for maintaining physiological benefits. In a recent review on supportive interventions designed to improve QoL for men with PCa, FACT-P was most frequently used to measure QoL [39]. Other instruments, such as the EORTC Quality of Life Questionnaire–Prostate Module (QLQ-PR25) or generic health-related QoL measurements like the Short-Form 36 Health Survey, are possible alternatives. Our aim, however, is to employ a prostate-specific, validated and frequently used QoL instrument to enhance comparability and validity, which is why we have chosen to use FACT-P for the primary outcome.

Large trials examining exercise interventions for improving QoL in cancer patients have been shown to be at risk of bias in a number of domains [40], e.g. selection bias and attrition bias due to unclear allocation concealment and incomplete outcome data. It has been argued that one of the barriers preventing decisions-makers from supporting this kind of intervention is that exercise science is not of the same calibre as other fields of medical science [41]. Due to the nature of non-pharmacological interventions, blinding often proves impossible [42] but that does not prevent behavioural studies from adhering to the majority of the ICH-GCP principles. The present study will adhere to these principles in order to conduct a high trial with enhanced comparability with medical drug trials while simultaneously preventing outcome reporting bias and erroneous interpretations of post-hoc analyses.

**Fig. 4:**
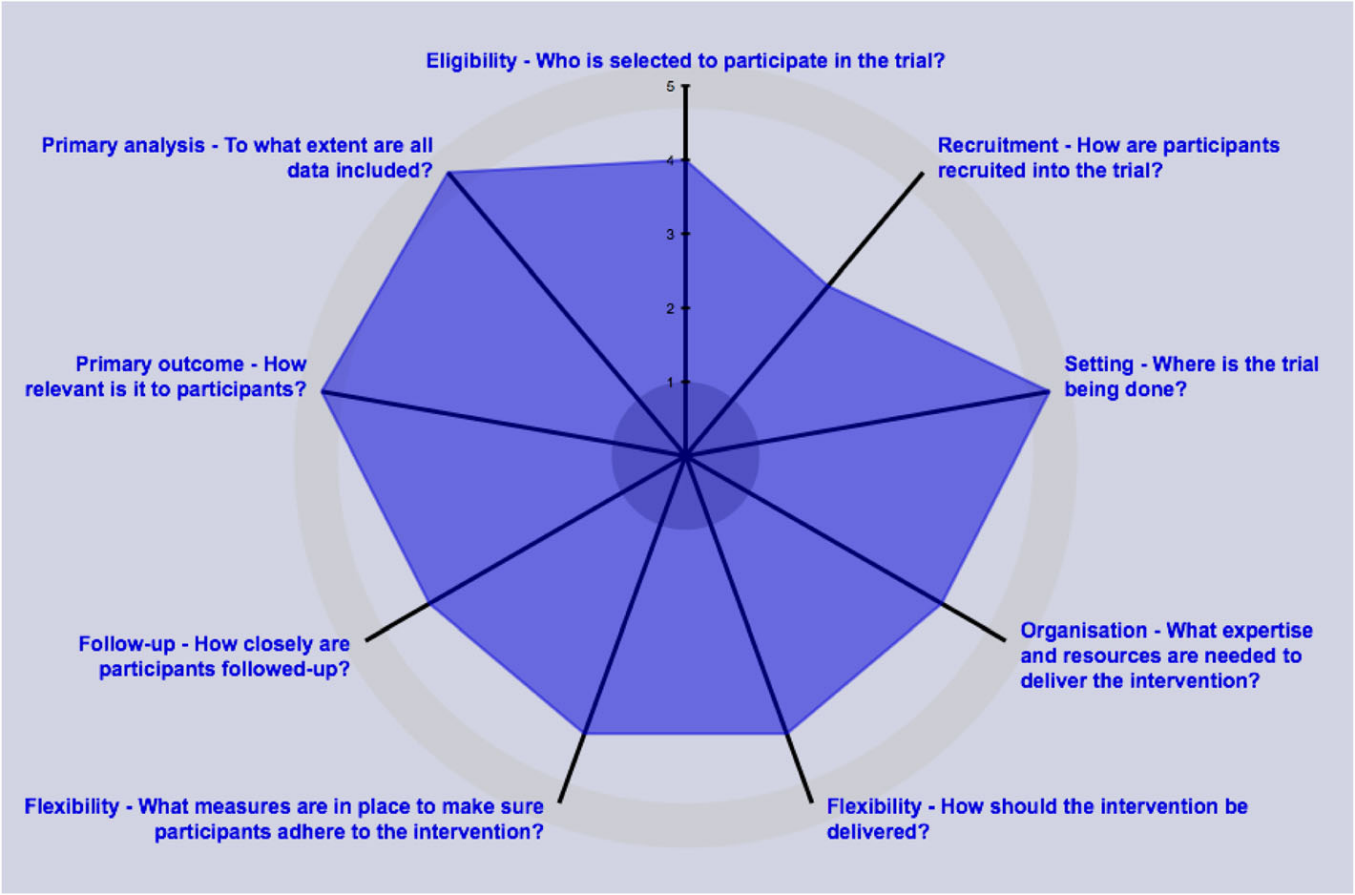
PRECIS-2 score of the FCPC Trial

A key aim of the FCPC Trial is to generate scientific knowledge to help support decisions on whether to use football in community-based clubs as a strategy for promoting health in men with PCa. For that reason the trial has utilised the PRECIS-2 wheel [43] to help define the pragmatic approach of the various domains, as shown in Fig. 4. The study was designed with this in mind and the recruitment of participants in multiple centres and delivery of the intervention in the community were chosen in order to enhance the generalisability of the results [26].

## Abbreviations

ADT: Androgen deprivation therapy
BMC: Bone mineral content
BMD: Bone mineral density
CHG-GCP: Council for Harmonisation Guideline on Good Clinical Practice
DAS–7: Seven-item Dyadic Adjustment Scale
DXA: Dual-energy X-ray absorptiometry
FCPC: FC Prostate Community Trial
IPAQ: International Physical Activity Questionnaire
LBM: Lean body mass
PCa: Prostate cancer
QoL: Quality of life
SF-12: 12-Item Short Form Health Survey
